# Alginate-modified graphene oxide anchored with lactoperoxidase as a novel bioactive nanocombination for colorectal cancer therapy

**DOI:** 10.1038/s41598-024-74604-0

**Published:** 2024-10-22

**Authors:** AbdElAziz A. Nayl, Esmail M. El-Fakharany, Ahmed I. Abd-Elhamid, Wael A. A. Arafa, Ahmed H. Alanazi, Ismail M. Ahmed, Mohamed A. Abdelgawad, Ashraf A. Aly, Stefan Bräse

**Affiliations:** 1 Department of Chemistry, College of Science, Jouf University, Sakaka, Aljouf 72341 Saudi Arabia; 2 Protein Research Department, Genetic Engineering and Biotechnology Research Institute GEBRI, City of Scientific Research and Technological Applications (SRTA city), New Borg El-Arab, Alexandria, 21934 Egypt; 3 Pharmaceutical and Fermentation Industries Development Centre (PFIDC), City of Scientific Research and Technological Applications (SRTA-City), New Borg El-Arab, Alexandria, Egypt; 4 Pharos University in Alexandria, Canal El Mahmoudia Street, Beside Green Plaza Complex, Alexandria, 21648 Egypt; 5 Composites and Nanostructured Materials Research Department, Advanced Technology and New Materials Research Institute, City of Scientific Research and Technological Applications (SRTA-City), New Borg Al-Arab, Alexandria, 21934 Egypt; 6 Department of Pharmaceutical Chemistry, College of Pharmacy, Jouf University, Sakaka, 72341 Al Jouf Saudi Arabia; 7 Chemistry Department, Faculty of Science, Organic Division, Minia University, El-Minia, 61519 Egypt; 8 Institute of Biological and Chemical Systems – Functional Molecular Systems (IBCS-FMS), Kaiserstrasse 12, Karlsruhe, 76131 Germany

**Keywords:** Graphene oxide nanocomposite, Biocompatible drugs, Alginate, Lactoperoxidase nanocombination, Anti-cancer and apoptosis, Biochemistry, Chemical modification, Enzymes

## Abstract

It is imperative to explore new biocompatible drugs with low toxicity for use in medicinal fields such as fighting tumors. Bovine lactoperoxidase (BLPO) stems from the most important enzymes in the bovine whey that provide a proper pattern for nano-formulation with nanomaterials. LPO is a suitable protein to be coated or adsorbed to alginate modified graphene oxide (GO-SA), which forms the modified GO-SA-LPO hybrid structure. This novel combination provides LPO stability with strong anticancer effects and boosts immunity response. The characterization results obtained from different techniques confirmed a successful LPO adsorption on the GO-SA composite surface. Moreover, nano-formulation of LPO with GO-SA composite exhibited a reduction in its size and overall charge. In addition, the experimental results showed greater LPO activity stability in the modified GO-SA-LPO nanocombination than free LPO after storage for 10 weeks at 4 °C. The in vitro study, a crucial step in the validation of our approach, demonstrated that the modified GO-SA-LPO nanocombination showed a potent anticancer selectivity toward colon cancer cell lines more than GO-SA composite or free form of LPO, which enhanced in a dose-dependent manner with high safety manner against normal cells. The apoptotic effect of this novel nanocombination was confirmed by the greatest variations in the expression of both well-known apoptosis genes (p53 and Bcl-2), severe changes in the cellular morphology, DNA fragmentation, and nuclear staining with fluorescence yellow and orange of the target cancer cells. Also, this superior efficacy of the modified GO-SA-LPO nanocombination was induced by suppressing some pro-inflammatory cytokines, including tumor necrosis factor-alpha (TNF-α), interleukin (IL-6), and necrosis factor-kappa B (NF-ĸB). Our observations presented that the modified nanocombination of LPO may offer a novel remedy for treating colon tumors *via* induced apoptosis pathway, inflammation reduction, and immune response improvement.

**Supplementary Information:**

The online version contains supplementary material available at 10.1038/s41598-024-74604-0.

## Introduction

The development in tumor mortality rates worldwide, especially in developing countries, is owing to many reasons, including aging, smoking, stress, environmental pollution, lack of physical activity, and radiation^1^. Colorectal cancer is one of the highest causes of morbidity and mortality rates worldwide. It is considered the third most common cause of death and equally affects both men and women, with incidences of more than 1.9 million and 0.9 million deaths worldwide recorded in 2020^2^. Although chemotherapy considerably enhances the fatigue of patients with colorectal tumors, only mild progress in survival rate can be accomplished owing to drug resistance and poor bioavailability. Subsequently, there is a great challenge to discovering a complete remedy for colorectal tumors. Several nanocomposites have been developed for cancer therapy as novel drug carriers and candidates to overcome this.

Graphene oxide (GO) is an adorable material composed of a single carbon layer with one C-atom thickness. Oxygenated function groups (epoxide, hydroxyl, carbonyl, and carboxylic) decorated the surface and the edges of the GO layer. Due to the previous promising properties (function groups and *π*-electrons localized on the GO-layer), the GO can interact covalently with cancer-cell targeting antibodies for employment in targeted drug delivery and noncovalent interactions with aromatic antitumor drugs^3,4^. Moreover, the presence of GO can improve the formation of reactive oxygen species (ROS) in cells, which explains the toxicity effect of most nanomaterials^5,6^. These ROS species can attack the cell’s biological macromolecules, such as proteins, cell membrane lipids, deoxyribonucleic acid (DNA), and ribonucleic acid (RNA), leading to numerous signal transduction pathways. This will result in inflammation, malignant transformation, proliferation, and apoptosis^7^. Hence, the GO has dual functions, such as carrying drugs and inducing inhibitory effects on tumor cells. The oxidation ratio of the GO plays an important role in the GO anticancer property. For example, a GO nanolayer with oxygen density (C/O ratio of 2.8: 1) could be applied to oxidative stress, cytotoxicity, and pulmonary toxicity. On the other hand, the GO, which has a low oxygen concentration (C/O ratio of 3.1: 1), leads to higher infiltration, interaction, and clearance of immune cells following both subcutaneous and peritoneal implantation^8^.

Several trials were investigated to use GO as a drug carrier or modify its nanolayer to improve its anticancer activity, such as the decoration of GO with silver nanoparticles (Ag NPs) and applying vitamin C for reduction. The results showed that the reduced GO-Ag NPs presented improved cytotoxicity activity with an IC_50_ value of (30 µg/mL) against lung A549 cancer cells, which is larger than that of GO (180 µg/mL), and importantly, the embryotoxicity of Ag NPs was significantly reduced by after decoration with reduced GO, indicating the biocompatibility of as-synthesized nanocomposite^9,10^. A composite platform composed of graphene oxide/chitosan and xyloglucan (GO-CH-Xn) was investigated for the assembling and liberation of doxorubicin (DOX). The prepared GO-CH-Xn-DOX shows good anticancer ability against human (U 87) glioblastoma cancer cell lines^11^. A previous study used GO/poly(ethylene glycol)-b-poly(2-hydroxyethyl methacrylate-g-lactide)_2_ nanocomposites (GO/PEG-b-poly(HEMAg-LA)) and loaded with DOX for anticancer applications with safe manner^12^. Another study used loaded GO with the chemotherapeutic drug cisplatin (CP) to produce GO-CP complex and examined its anticancer activity against human lung A549 cancer cells^13^. In addition, GO had been modified with chitosan (Chr) to form ChrGO nanosheets using microwave and immobilized by Adriamycin (ChrGO/Adriamycin), which were found to exhibit anticancer activity against human ductal breast cancer (BT-474) cells^14^. A successful modification of superparamagnetic graphene oxide (GO–Fe_3_O_4_) nanohybrid with 3-aminopropyl triethoxysilane (APS) modified and anchored with DOX exhibited advanced use for targeted delivery and limited release of anticancer drugs evaluate their targeted delivery character and toxicity to tumor cells^15^. Yaghoubi et al.^16^ revealed that aptamer-carboxylated graphene oxide (APT-CGO) involving combined anticancer drugs curcumin and DOX displayed a thermo- and pH-sensitive drug release behavior that exhibited a great anticancer affinity against human gastric adenocarcinoma (AGS) cell line and minimized the expression of AKT, CDK2, and NF-KB. At the same time, it induced RB1 expression at the protein and gene levels. Pie et al.^17^ revealed that the modified PEGylated nano-graphene oxide (pGO) was successfully loaded with dual chemotherapies of cisplatin and doxorubicin to promote interacted chemotherapy in one system. Authors demonstrated that the prepared system of loaded pGO with cisplatin and DOX prompted the great prominent cancer cell apoptosis and necrosis rate (18.6%), which was 2 folds larger than that of pGO-cisplatin or pGO-DOX systems^17^.

Bovine lactoperoxidase (LPO) is a glycoprotein with a covalently bonded heme prosthetic group in its catalytic core. LPO was recovered in its crystalline form from skimmed milk and subsequently isolated from various exocrine secretions, including tears, saliva, and airways^18^. Furthermore, other glands, including the salivary, hardenian, and lacrimal glands, release this enzyme^19,20^. Bovine milk LPO is a member of the peroxidase enzyme family, primarily responsible for primary control catalysis in the oxidation of certain molecules. In the presence of hydrogen peroxide (H_2_O_2_), all peroxidases catalyze a similar oxidation reaction *via* several stages, producing various strong products with a broad range of biological and antibacterial activity^21^. LPO acts as an antibacterial agent *via* the LPO system (LPS), a particular inhibitory mechanism of the presence of H_2_O_2_ and thiocyanate ions (SCN^−^). Furthermore, LPS plays a critical role in the innate immunity system, which may play a key role in the breakdown of some toxins like aflatoxin, as its effects remain untouched by antimicrobial activity^22^. Therefore, considering the current state of technological development, NPs might be a good choice for boosting and stabilizing LPO activity for various applications. Because of the multiple roles of LPO, this enzyme perceives an active target as being coated or encapsulated in NPs to form unique nano-combinations with controlled surface features. The discovery of approaches that could improve conformational stabilization for several weeks of LPO *via* nanoformulation should promote the applicability of this bioactive ingredient in biopharmaceutical applications. A previous study^23^ demonstrated that silica NPs can modify the conformation of LPO when it adsorbs on their surface. Consequently, new biotechnological capacities were improved, such as the ability to kill various types of multi-resistant microbes24.

In this work, we modified the GO nanosheet with alginate (SA) biomaterial to form the modified GO-SA nano-composite. After that, the purified LPO was immobilized on the modified GO-SA nano-composite to form a novel nano-combination of GO-SA-LPO as a potent anticancer agent against colon cancer as an in vitro study. The modified GO-SA-LPO nano-combination was characterized by SEM, TEM, FTIR, TGA, Raman spectroscopy, and zeta potential techniques. The stability of the purified LPO before and after conjugation with the modified GO-SA nanocomposite was investigated. Moreover, the anticancer activity of free LPO, the modified GO-SA nanocomposite, and the modified GO-SA-LPO nano-combination against normal human lung fibroblast WI-38 cells and both colon (Caco-2 and HCT-116) cancer cell lines were also investigated.

## Materials and methods

### Chemicals, materials, and instruments

All chemicals, materials, and instruments used in this work were explained and discussed in the Supplementary Material file (Sect. 2.1.).

### Purification of bovine milk lactoperoxidase

Bovine milk lactoperoxidase (BLPO) was extracted from bovine skimmed milk. First, the milk fat was removed by centrifugation at high speed for 1/2 hour. After that, defatted milk was decaffeinated by lowering the pH to 4.2 with 1 M HCl. The obtained skimmed milk was dialyzed against 50 mM phosphate buffer, pH 7.2, and about 100 mg protein was applied into Mono S 5/50 GL column pre-equilibrated with 50 mM phosphate buffer, pH 7.2. The bound proteins were eluted with a stepwise gradient of 0.0 to 1.0 M NaCl prepared in 50 mM phosphate buffer, pH 7 at a 1 mL/min flow rate and 4 mL fractions. All fractions of LPO were concentrated and desalted separately by a centricon ultrafiltration cell (30 kDa MWCO). The purification process of LPO investigated in this study was explained in the Supplementary Material file (Sect. 2.2.).

### Preparation of the modified GO-SA composite

The GO-SA was fabricated in our previous work^24^. In brief, 3.0 g graphite (SA) was stirred with 70 mL conc. H_2_SO_4_, and 9.0 g KMnO4 were gradually added to the previous mixture in an ice bath over 1 h. After that, the obtained blend was added to 500 mL H_2_O and stirred for another 0.5 h. Fifteen mL H_2_O_2_ (30%) was added to form a yellow precipitate, which was filtered, washed with 10% HCl solution, and three times with water to obtain GO composite. The obtained solid was air-dried for further use. To synthesize the modified GO-SA composite, 0.15 g of the obtained GO composite was resuspended in distilled water and mixed with SA solution (2% wt) at constant stirring to form a uniform GO-SA mixture solution. After that, tetraethylorthosilicate solution (5 mL tetraethylorthosilicate to 25 mL absolute ethanol) was added to the prepared GO-SA mixture and stirred for 48 h at 60 °C to form the modified GO-SA composite. The obtained solid centrifuge was washed with ethanol and distilled water, lyophilized, and kept at 4 °C until further use.

### Preparation of the modified GO-SA-LPO nanocombination

The lyophilized GO-SA composite was resuspended in 50 mM Tris HCl buffer, pH 7.2, and sonicated for 1 min. For modification of the surface of the prepared GO-SA composite, LPO (0.1 g/ml) was added dropwise to GO-SA composite (0.2 g/ml) in 0.1 M phosphate buffer (pH 7.2) with continuous stirring for 2 h. Then, the modified GO-SA-LPO composite was separated by centrifugation at 4000 xg (Biofuge primoR, Heraeus, Germany) for 30 min at 4 °C. The obtained residue was washed three times with phosphate buffer saline (PBS), freeze-dried, and kept at -4 °C until further use^25^. The total protein content was assayed using the Bradford method to determine protein concentration in the supernatant and confirm that all used amounts of LPO were modified with GO-SA composite.

### Characterization of the modified GO-SA-LPO nano-combination

The fabricated of the modified GO-SA and GO-SA-LPO composites were characterized by a Scanning Electron Microscope (SEM) (JEOL GSM-6610LV. Japan). Transition Electron Microscope (TEM) analysis was carried out by a JEM-1010 unit (JEOL Ltd., Tokyo, Japan) with an additional EDS unit, Fourier Transmission Infra-Red Spectroscopy (FT-IR) (8400s, Shimadzu, Japan) covered the range from 400 to 4000 cm^−1^ and Raman Spectroscopy (Bruker, Senterra II, Germany) and Thermo-Gravimetric Analysis (TGA, Shimadzu Thermal Gravimetric Analysis (TGA)—50, Japan) and Raman Spectroscopy (Bruker, Senterra II, Germany).

### Determination of LPO activity and stability

The activity of the purified LPO as a free form and nano-formulated form (GO-SA-LPO) was assayed as described by Chance and Maehly^26^. In this method, the activity of LPO was estimated based on the ability of the LPO/H_2_O_2_ system to oxidize guaiacol to yield tetraguaiacol (a brownish-orange colored product), which was detected at 470 nm against the times every 15 s for 3 min. To determine the stability of the purified LPO and nano-formulated form, the LPO activity in both free and nano-formulated forms was assayed during storage conditions throughout 10 weeks at 4 °C^27^. One international unit (IU) of LPO activity was identified as the LPO concentration that converted 1.0 µmol guaiacol to produce tetraguaiacol per min using a coefficient of 26.6 mM^−1^cm^−1^.

### In *vitro *assaying anticancer activity

#### Cell culture and media

Human normal lung fibroblast (WI-38), human colorectal adenocarcinoma (Caco-2), and (HCT-116) cell lines were obtained from the American Type Culture Collection, USA. DMEM and RPMI-1640 media were purchased from SERANA Co. (SERANA, Germany), and fatal bovine serum (FBS) was purchased from Gibco Co. (Gibco, US). Human WI-38 and Caco-2 cell lines were cultured and maintained in DMEM media supplemented with 10% FBS. However, HCT-116 cells were cultured and maintained in RPMI-1640 media supplemented with 10% FBS.

### Cytotoxicity of the modified GO-SA-LPO nano-combination

The antitumor properties of the functionalized GO-SA composite and the modified GO-SA-LPO composite against both normal and colon tumor cell lines were investigated using MTT (3-[4, 5-Dimethylthiazol]-2, 5-Diphenyltetrazolium bromide) assay as described in our lab by Mosmann^28^. In Brief, all cell lines (1.0 × 10^4^/well) were seeded in three sterile 96-well microplates overnight at 37 °C in supplemented media with 10% FBS. Both normal and tumor cell lines were exposed to the GO-SA composite and the modified GO-SA-LPO composite at different concentrations of 12.5, 25, 50, 100, 200, and 400 µg/ml in triplicates. Cytotoxicity of the modified nanocomposites was evaluated as discussed in the Supplementary Material file (Sect. 2.7.2.).

### Nuclear staining

The antitumor effect of the prepared GO-SA composite and the modified GO-SA-LPO composite against Caco-2 cells was investigated by using methods of one fluorescent stain using propidium iodide (PI) dye and double fluorescent stain using acridine orange and ethidium bromide (AO/EB) dyes. Briefly, the treated Caco-2 cells for 48 h were washed twice with fresh media and fixed with 4% paraformaldehyde for 10 min at 37 °C. The cells were permeabilized with 0.5% Triton X-100 in 3% paraformaldehyde for 1 min, then PI and AO/EB dyes (10 µg/ml in PBS, pH 7.2) were added separately, and the cells were incubated for 20 min in a dark condition. The stained cells were washed with PBS and visualized under a fluorescence phase contrast microscope (Olympus, Japan) using an excitation filter of 480/30 nm.

###  Effect of the modified GO-SA-LPO composite on gene expression

The effect of the prepared GO-SA composite and the modified GO-SA-LPO composite on the expression level of tumor suppressor gene (p53) and oncogene (Bcl-2) was determined in human Caco-2 and HCT-116 cells using the qPCR method and explained in the Supplementary Material file (Sect. 2.8.).

#### Effect of the modified GO-SA-LPO composite on pro-inflammatory markers

Both treated Caco-2 and HCT-116 cells were lysed by RIPA lysis buffer for assaying protein expression levels. Total protein concentrations in all tested samples were estimated using Bradford’s method (1976). According to their manuals, the protein expression levels of pro-inflammatory markers TNF-α, IL-6, and NF-ĸB were estimated using ELISA reagent kits (Sunlong Biotech Co, Ltd, China).

### Statistical analysis

Data were expressed as mean ± standard deviation (SD). The multiple comparisons determined statistical significance, and the obtained data were estimated by the multiple comparisons Tukey post-hoc analysis of variance (ANOVA) using the SPSS16 program. The differences were considered statistically significant at *p* < 0.05.

## Results

### Purification of bovine lactoperoxidase

Bovine LPO was extracted from bovine skimmed milk (whey) by defatting and decaseination using two simple steps. For the first step, whey proteins were applied into a cation exchange Mono S 5/50 GL column, and LPO was eluted from the column using a stepwise gradient of 0.0–1.0 M NaCl. For the second step, the fractions from the Mono S 5/50 GL column, which showed the activity of LPO, were applied into the gel filtration Sephacryl S-100 column for further purification of LPO. All fractions showing positive LPO activity were collected, lyophilized, and kept at -20 °C for further use.

### Synthesis and characterization of the modified GO-SA-LPO composite

**Figure 1:**
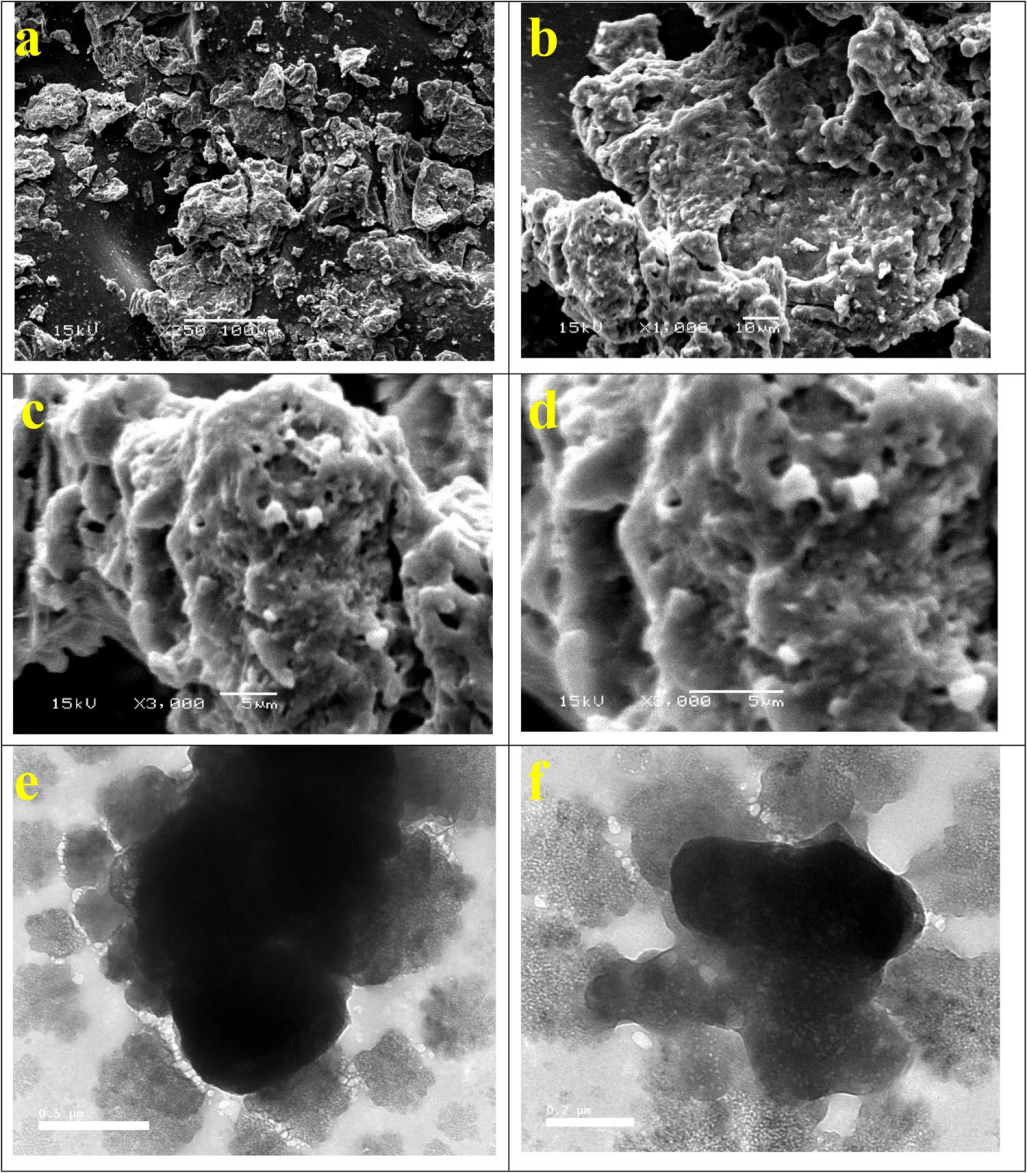
Characterization of the modified GO-SA-LPO composite Scanning electron microscopy (SEM), transmission electron microscopy (TEM). (**a**-**d**) SEM images of the modified GO-SA-LPO composite at different magnifications 250, 1000, and 3000X and (**e**-**f**) TEM images of the modified GO-SA-LPO composite.

**Figure 2:**
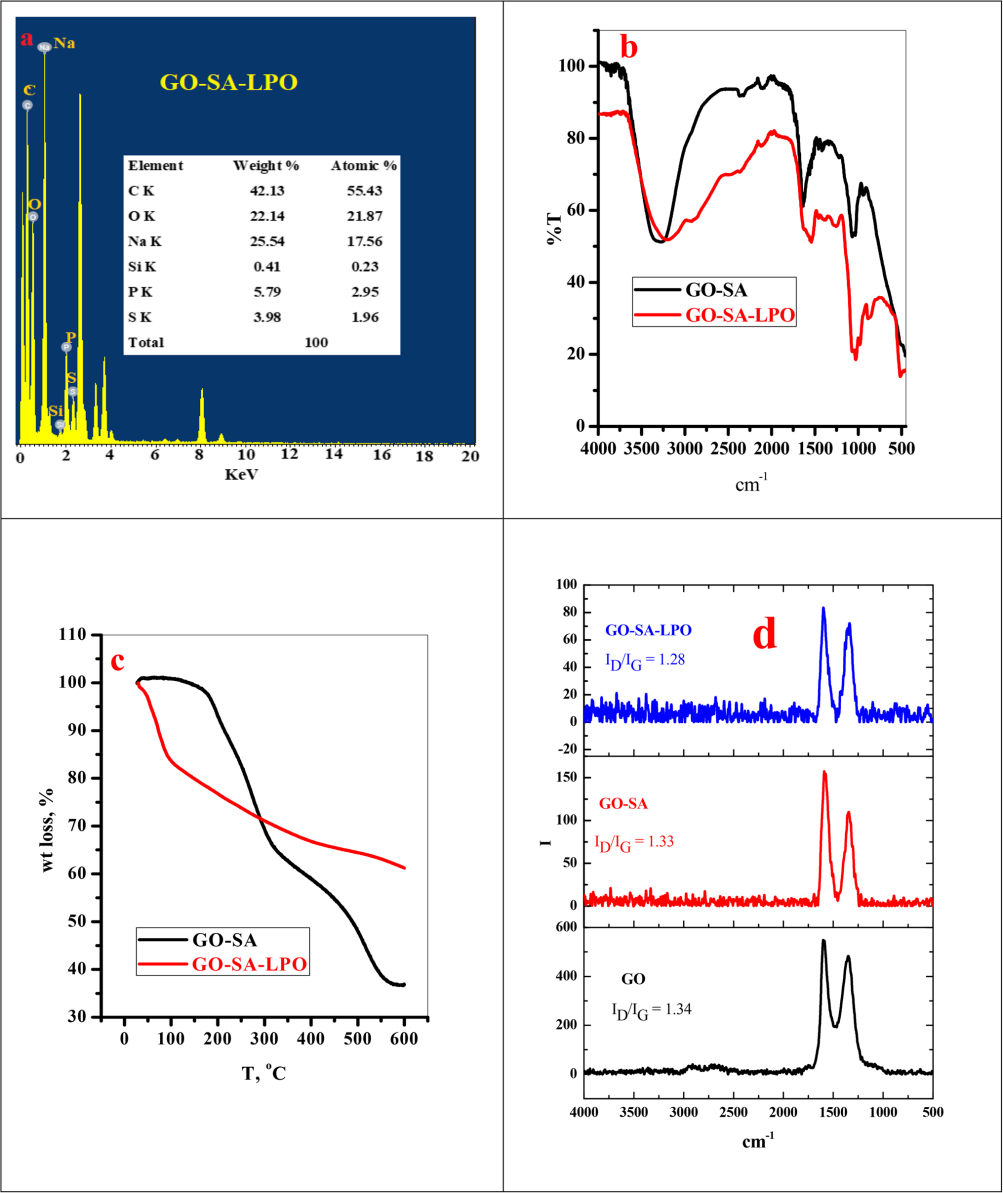
(**a**) TEM-EDS analysis of GO-SA-LPO composite, (**b**) FTIR, (**c**) TGA and (**d**) Raman of GO-SA and GO-SA-LPO composite.

The SEM images of the GO-SA composite appeared as a single layer with alginate deposits on its surface, as observed in our previous work^24^. Upon treating the prepared GO-SA composite with LPO, the produced composite presents some agglomeration, possibly due to the complexion reaction between the GO-SA composite and LPO, as shown in Fig. 1 (a-d). Moreover, the TEM photograph of the GO-SA composite shows that this composite appeared to be a dull layer. By treating the GO-SA with LPO, we observed the presence of the cloudy GO-SA composite layer surrounded with transparent materials, which may be referred to as the LPO, refer to Fig. 1 (e-f). The GO-SA composite consists of C, O, Si, and Na. On the other hand, the modified GO-SA-LPO composite shows the same composition plus the presence of P and S, which may confirm the assembly of LPO on the GO-SA composite surface, see Fig. 2a.

**Figure 3:**
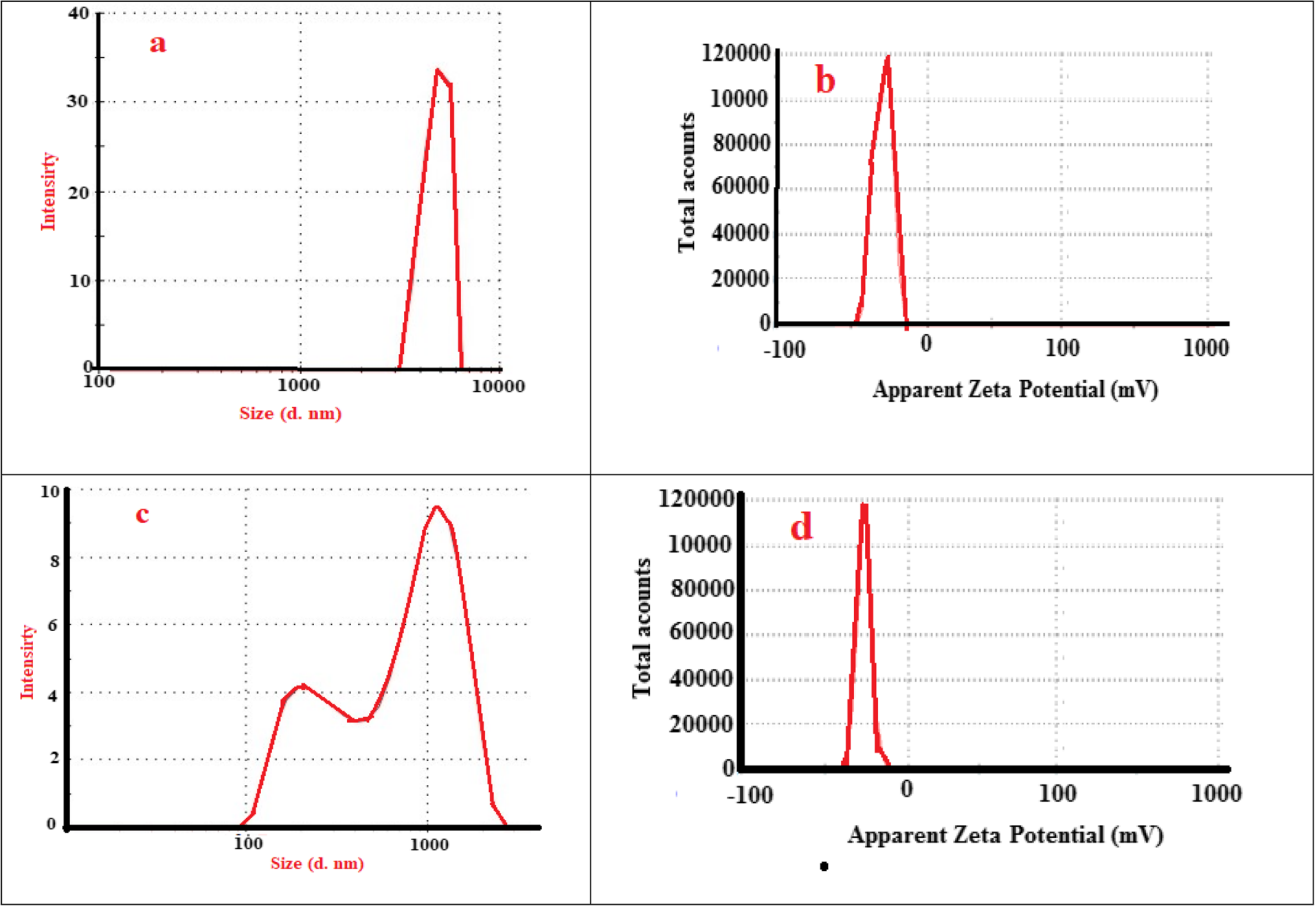
(**a**, **c**) Particle size analysis and **b**, **d**) zeta potential of GO-SA and GO-SA-LPO composite.

The FTIR analysis of GO-SA shows four main peaks at 1726 cm^−1^ (COO^−^), 1602 cm^−1^ (bending motion of the water molecule), 1406 cm^−1^ (C = O deformation), and 1029 cm^−1^ (stretching motion of C-O and Si-O bonds) see Fig. 2b. The immobilization of LPO to the GO-SA leading to the appearance of a new peak at 3207 cm^−1^ (O-H stretching) and 2915 cm^−1^ (C-H stretching), 1541 cm^−1^ (N-H stretching related to LPO) and 1458 cm^−1^ (C-N stretching) as shown in Fig. 2b. And disappearance of the peaks at 1726 and 1602 cm^−1^. The TGA analysis is required to assess the thermal stability of the analyzed material. Here, the loading of LPO on the GO-SA composite surface will lead to enhance the burning stability of the GO-SA-LPO composite than the original material, as shown in Fig. 2c. The particle size analysis showed that the addition of LPO to GO-SA will reduce the particle size and the negative charge of GO-SA-LPO composite than GO-SA, as presented in Fig. 3. This is due high pairing between the negative GO-SA and positive LPO which result in reducing the large GO-SA layer to low size agglomerated structure.

Raman spectra are a vital tool for analyzing graphitic materials. This device provides excellent information about the defects in the carbonous structure. Raman spectra of GO, GO-SA, and GO-SA-LPO are explained in Fig. 2d; Table 1. Typically, as illustrated in our previous report^24^, the modification of GO with SA caused a shift in the G-band (which is related to vibration of sp2-C) from 1598 to 1584 cm-1 and the D-band (characterized by the sp^3^-C) from 1352 to 1335 cm^−1^. This may be due to the loading process carried out through a covalent bond with the O-groups spread over the GO surface. After loading the LPO over the GO-SA, the I_D_/I_G_ values small changes which suppose that the LPO loading process is not influenced by the electronic structure of the GO-SA nanomaterials.

**Table 1:** The D and G-band positions, I_D_/I_G_ ratios, and FWHMs.

Sample	D-band peak		G-band peak		I_D_ /I_G_
	Raman shift (cm^-1^)	FWHM (cm^−1^)	Raman shift (cm^−1^)	FWHM (cm^−1^)	
GO	1352	150	1598	112	1.34
GO-SA	1335	100	1584	75	1.33
GO-SA-LPO	1337	86	1600	67	1.28

### Stability of the nanoformulated bovine lactoperoxidase

The stability of LPO in both nanoformulated and free forms was evaluated by estimating the enzyme activity within 10 weeks of storage at 4 °C in the refrigerator. The free form of LPO steadily lost its activity through storage activity by increasing the storage time to 0 IU at week 8 (Table 2). However, the nanoformulated form of (GO-SA-LPO composite) was more stable during the storage condition, and its activity became around 31% of its starting activity at week 10 of the storage period (Table 2). Consequently, our findings reveal that LPO activity in nanoformulated form (GO-SA-LPO composite) was more stable with half-life time of 4 weeks compared to free LPO after storage.

**Table 2:** Stability of the purified bovine LPO activity (IU) as free and nanoformulated (GO-SA-LPO composite) forms within 10 weeks of storage at 4 °C.

Week	1	2	3	4	5	6	7	8	9	10
LPO	64.67 ± 3.06^a^	48.67 ± 2.08^ab^	29.33 ± 2.52^ab^	9.26 ± 0.75^ab^	4.69 ± 0.27^b^	1.65 ± 0.13^b^	0.45 ± 0.05^b^	0	0	0
GO-SA-LPO	64.33 ± 3.21^a^	57.67 ± 3.51^a^	52.33 ± 3.05^a^	46.67 ± 2.08^a^	44.17 ± 2.52^a^	40.33 ± 3.06^a^	38.17 ± 2.75^a^	35.16 ± 2.25^a^	33.67 ± 2.52^a^	30.67 ± 1.53^a^

All results are represented as mean ± SD, and letters differ significantly within the same raw at *p* < 0.05.

### Anticancer activity of the modified GO-SA-LPO NPs

**Figure 4:**
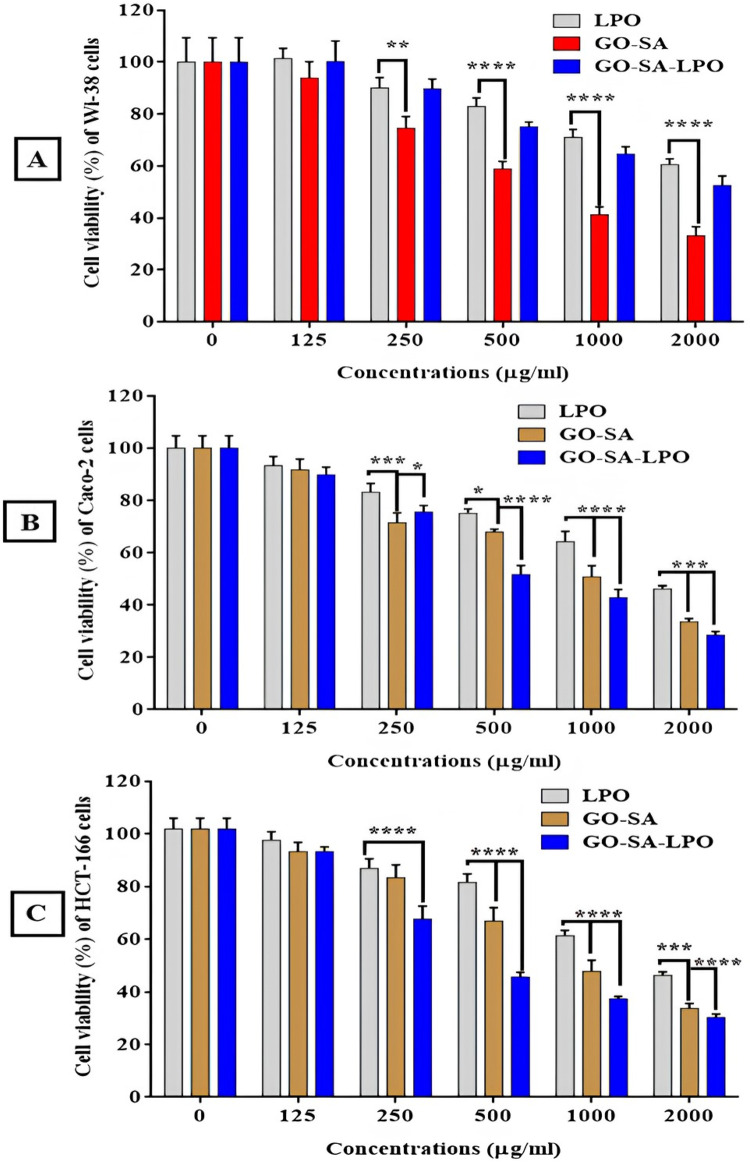
The cell viability profile of the treated Wi-38 cells (**A**), Caco-2 cells (**B**), and HCT-116 cells (**C**) with the prepared GO-SA NPs and the modified GO-SA-LPO NPs in comparison with LPO. All treated cells were incubated with the prepared and modified NPs at different concentrations (125, 250, 500, 1000, and 2000 µg/mL)/48 h. All data are represented as mean ± SD and represent the average values from three experiments (*n* = 3), and data were considered highly vary at *p* < 0.05 *, *p* < 0.005 **, *p* < 0.0005 ***, *p* < 0.0001 ****.

**Figure 5:**
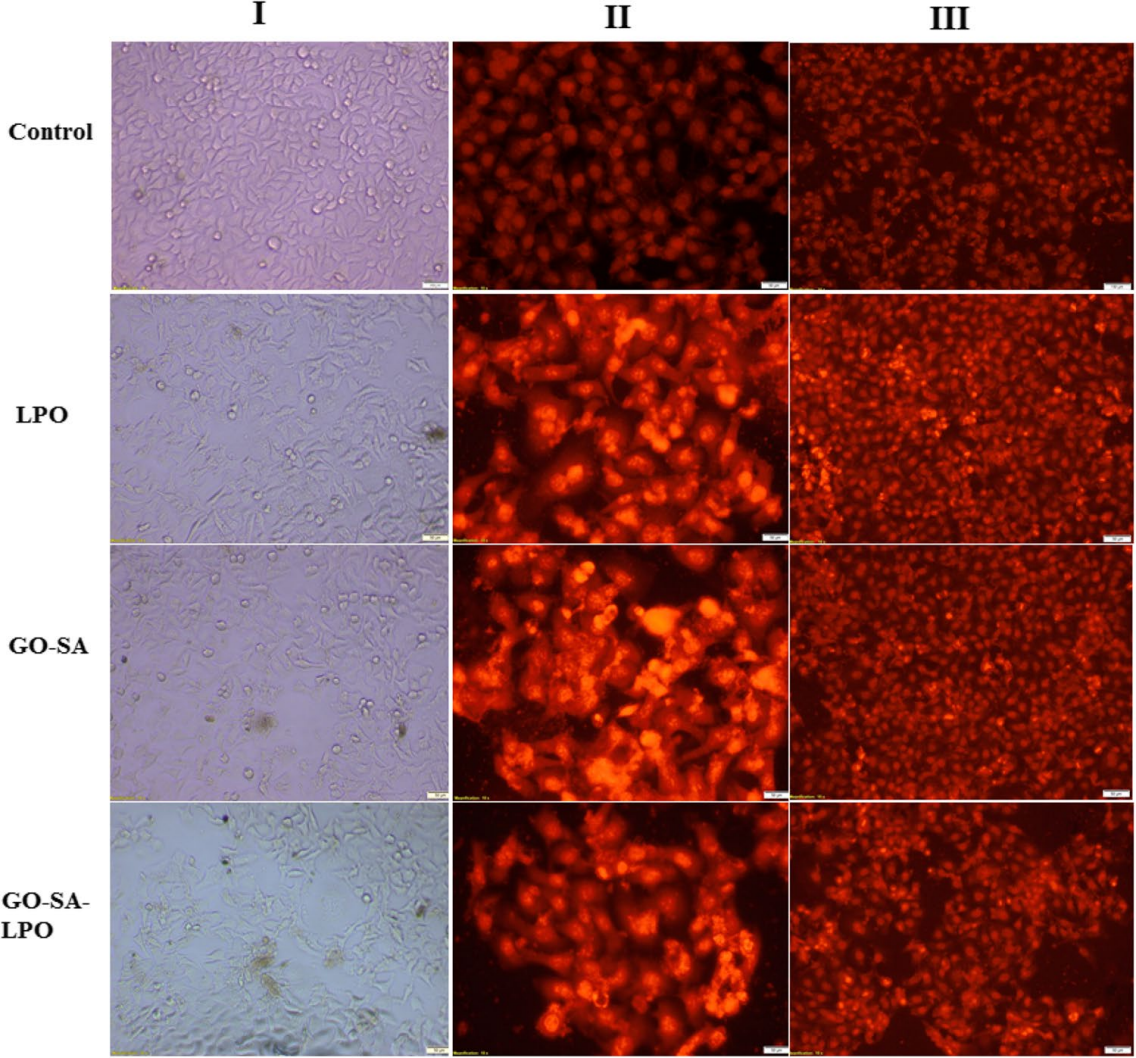
Effect of the prepared GO-SA composite and the modified GO-SA-LPO composite on the morphological and nuclear changes of Caco-2 cells compared to free LPO. (I) Micrographs of the treated Caco 2 cancer cells are shown under an inverted phase contrast microscope. (II and III) Investigate the apoptotic effect of LPO, GO-SA composite, and the modified GO-SA-LPO composite using PI fluorescence staining and double fluorescence staining (AO-EB) of Caco-2 cells, respectively. Caco-2 cells were exposed to the IC50 value of each prepared NPs for 48 h, and untreated (control) cells were included as control cells.

In the current in vitro study, GO-SA NPs and the modified nanoformulation of GO-SA composite with LPO were evaluated for mediating a selective apoptotic effect on the treated colon cancer cells for 48 h. It was observed that the modified GO-SA-LPO composite showed a potent anticancer ability against colon cancer cell lines more than the GO-SA composite or free of LPO. In general, our results indicated that the cytotoxic effect of the prepared GO-SA-LPO composite on the human normal Wi-38 cell line was less than their effect on the tested colon cancer cell lines. Remarkably, the modified GO-SA-LPO improved their anticancer activity and selectivity against colon cancer cell lines. The IC_50_ values were found to be decreased for GO-SA-LPO composite (< 296.8 µg/ml) than LPO (> 1116 µg/ml) and GO-SA composite (> 620.3 µg/ml), as shown in Table 3. Interestingly, the modified GO-SA-LPO composite showed safe (EC_100_) values of 21, 32, and 28 µg/ml with the highest selectivity (SI) values of 3.69 and 4.18 against both Caco-2 and HCT-116 cells, respectively (Table 3). More significantly, the modified GO-SA-LPO composite exerted a notable enhancement in the anticancer ability against the treated Caco-2 and HCT-116 cells in a dose-dependent manner with a high safety profile against normal Wi-38 cells more than treated cells with GO-SA composite (Fig. 4). Consequently, the morphology of treated-Caco-2 cells was found to be more changed to the modified GO-SA-LPO composite more than treatment with LPO or GO-SA composite. Figure 5I indicates the stimulation of morphological modifications with cell destruction in the treated-Caco-2 cells that combined with shrinkage and nuclear condensation compared to untreated (control) cells.

**Table 3:** EC100, IC50 (µg/mL), and SI values of the modified GO-SA-LPO composite against Wi-38, Caco-2, and HCT-166 cell lines after treatment for 48 h compared with free LPO and GO-SA composite.

Cell lines		LPO	GO-SA composite	GO-SA-LPO composite
Wi-38	EC_100_	53 ± 2.44	18 ± 1.17	46 ± 2.65
	IC_50_	1350 ± 52.13	792.4 ± 57.54	1097 ± 59.68
Caco-2	EC_100_	59 ± 3.58	15 ± 1.39	23 ± 1.74
	IC_50_	1488 ± 24.01	606 ± 10.74	296.8 ± 4.24
	SI	0.91 ± 0.04	1.31 ± 0.09	3.69 ± 0.21
HCT166	EC_100_	48 ± 3.07	16 ± 1.23	21 ± 1.93
	IC_50_	1116 ± 33.62	620.3 ± 7.33	262.7 ± 5.42
	SI	1.21 ± 0.05	1.28 ± 0.09	4.18 ± 0.23

All data were expressed as mean ± SEM.

**Figure 6:**
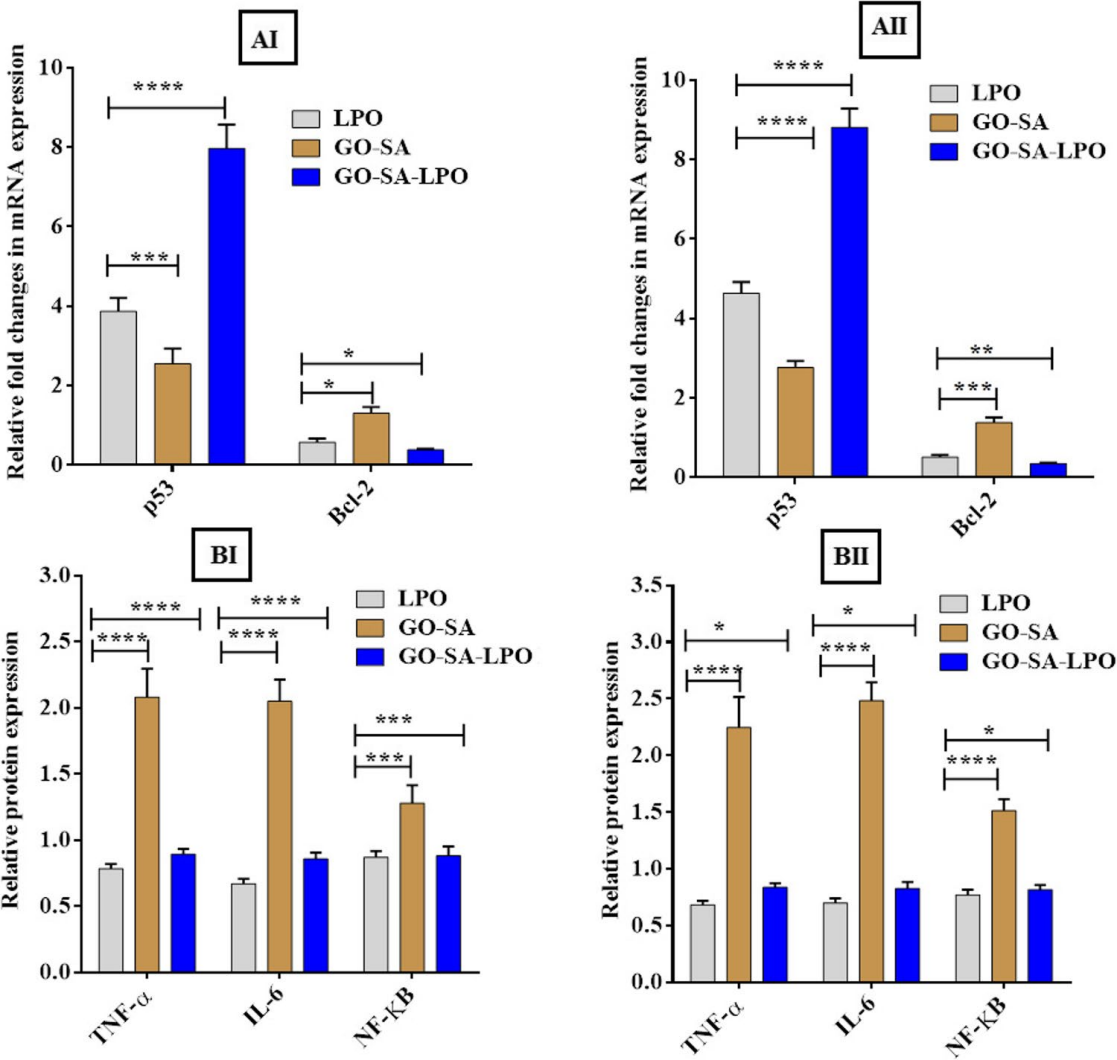
Apoptotic effect of the modified GO-SA-LPO NPs against Caco-2 cells and HCT-116 cells. (**A**)The relative fold change of gene expression levels of p53 and Bcl-2 in the treated Caco-2 cells (I) and HCT-116 cells (II) with the prepared GO-SA and the modified GO-SA-LPO composites compared with free LPO for 48 h. (**B**) The effect on the protein expression of TNF-α, IL-6, and NF-ĸB in Caco-2 cells (I) and HCT-116 cells (II) after treatment for 48 h with the prepared GO-SA and the modified GO-SA-LPO composites in comparison with free LPO. Untreated cells were included as a control reference, and all values are demonstrated as mean ± SD. represent the average values from three experiments (*n* = 3) and data were considered highly vary at *p* < 0.05 *, *p* < 0.005 **, *p* < 0.0005 ***, *p* < 0.0001 ****.

On the other hand, a potent apoptotic property of the modified GO-SA-LPO composite was confirmed by capturing alternations in the morphology of the treated-Caco-2 cells at IC_50_ doses *via* nuclear staining method by PI dye and double (EB/AO) stain as shown in Fig. 5II & III, respectively. The stained Caco-2 cells with PI showed that their nuclei became more condensed and intensely fluorescent, combined with fragmented chromatins compared to control cells (Fig. 5II). Furthermore, staining the treated cells with EB/AO double stain indicated that both the modified GO-SA-LPO composite and GO-SA composite ascertained the incidence of apoptotic property at an early stage, which emitted a yellow fluorescence (Fig. 5III). Additionally, some treated cells ascertained the incidence of apoptotic property at a late stage, which was indicated by loss of cellular integrity and the fluorescence emitted was red and orange instead of green for control cells (Fig. 6B).

Interestingly, the expression level of the p53 gene exited up-regulated in both targeted Caco-2 and HCT-116 cells after treatment with the modified GO-SA-LPO composite. At the same time, the expression level of Bcl-2 was observed to be down-regulated in both treated cells (Fig. 6AI and AII). The expression levels of p53 in the targeted cells with the modified GO-SA-LPO composite were increased by more 8 folds than untreated control cells and about 3–4 folds than treated cells with GO-SA composite and free LPO. However, the expression levels of Bcl-2 in the treated cells with the modified GO-SA-LPO composite were found to be suppressed with more 2–3 folds than the untreated control cells and about 5 folds than treated cells with GO-SA composite (Fig. 6AI and AII). In addition, the apoptotic effect of the modified GO-SA-LPO composite on the expression of pro-inflammatory markers involving TNF-α, IL-6, and NF-ĸB in both treated Caco-2 and HCT-116 cells was estimated in comparison with GO-SA composite and free LPO. Our findings indicated that the modified GO-SA-LPO composite suppressed the expression level of TNF-α, IL-6, and NF-ĸB markers compared to untreated cells. However, in the treated cells with GO-SA composite, these markers’ expression level was slightly increased than in untreated cells (Fig. 6BI and BII).

## Discussion

Alginate (GO) is a copolymer in the linear form that consists of (1→4)-linked α-L-guluronic (G) and β-D-mannuronic (M) residues. Alginate is a biomaterial that naturally occurs in many tissues and organisms, such as brown seaweed and the capsule of bacteria such as *Pseudomonas* sp. and *Azotobacter* sp. Electrostatic repulsion among the charged groups on the polymer chain further contributes to the rigidity of the chains. Due to its favorable properties and biocompatibility, alginate is used in several applications in many fields, including biomedical science and engineering, textile, food, and paper industries^29^. The structural similarity of alginate hydrogels to the extracellular matrices in tissues might be impacted to play numerous critical roles, particularly in tissue engineering applications, wound healing, and drug delivery^30^. However, alginate has many limitations, such as gelation into softer structures when in contact with a physiological microenvironment, which controls its applications in soft tissue regeneration and becomes unsuitable for associating with load-bearing body tissues. Several materials and ingredients can be incorporated to alginate moieties to form new sturdy composites to overcome this shortage. For instance, adding natural and synthetic polymers, peptides, and proteins to the alginate structure produces an improved new composite material with enhanced mechanical features compared to the original alginate. Moreover, cell viability and drug release kinetic can be enhanced when alginate composite material is utilized as an encapsulating candidate^29^. Graphene oxide (GO) has greatly attracted attention to acting as a nanofiller to reinforce many polymers owing to its large theoretical specific surface area^31^. GO has a huge number of functional oxygen-containing groups, such as carboxyl, epoxide, and hydroxyl groups, which provide the hydrophilic property to GO, making it dispersible in water and easily modified to interact with polymer matrixes for improving the mechanical properties of hydrogel^30,31^.

The incorporation of GO with SA significantly enhances the tensile strength of the formed GO-SA composite hydrogel due to the homogenous dispersion of the GO nanosheet layers in the SA matrix environment^32^. Thus, upon preparation of the GO-SA composite through modification of the GO with SA, it still appears as a layer structure due to the repulsion force among the negative charges of the anionic carboxylate groups of the alginate. The LPO is a suitable protein for efficient interaction with the negative charges on the GO-SA composite surface owing to its cationic nature (carrying positive charges). LPO is a peroxidase-cyclooxygenase superfamily and one of the most vital enzymes in bovine milk. LPO has a molecular weight of approximately 78,000 Da and involves several carbohydrate groups^33^ and amino acids^34^. The mature amino acid structure of bovine LPO involves 15 cysteine residues that form 7 disulfide bonds (-S-S-): Cys6-Cys167, Cys15-Cys28, Cys129-Cys139, Cys133-Cys157, Cys237-Cys248, Cys456-Cys513, and Cys554-Cys579, whereas the remaining Cys441 retains as a free cysteine residue^35^. Bovine LPO is characterized by its highly cationic property with a pI of 9.6, and this high cationicity makes LPO have many significant roles in antimicrobial applications and biological activities^6,36^. By adsorption of LPO on the surface of the modified GO-SA composite, an efficient electrostatic interaction was expected to arise among the GO-SA and LPO to compose the modified GO-SA-LPO composite. Interaction between nanoparticles (NPs) and proteins leads to new NP-protein complexes called the corona, which originates from NPs bio-reactivity. This formed corona can lead to many changes in the structure of the interacted proteins, which might modify the total bio-reactivity of the NPs.

Additionally, the interaction between the NPs and protein may alter the protein structure and, consequently, may cause variation in the main function of the adsorbed protein^37^. In this investigation, we exposed the interaction of LPO with the prepared GO-SA composite to yield the modified GO-SA-LPO complex, which increases the retention of LPO and enhances its application as an anticancer candidate for treating many types of cancer cell lines. Due to this interaction, the GO-SA-LPO composite presents a degree of agglomeration, as proved by SEM results. Moreover, the TEM images of GO-SA-LPO appear as a cloudy layer of GO-SA surrounded by the LPO molecules. The GO-SA composite was prepared by blending GO and SA in the exitance of TEOS as a crosslinker. Therefore, the elemental composition of GO-SA is mainly composed of C, O, Na, and Si. After the interaction of LPO with the prepared GO-SA composite, new elements such as S and P were observed in the TEM-EDS elemental analysis of the produced composite GO-SA-LPO. The LPO molecule contains a heme group in its catalytic center attached by a disulfide bond to the single peptide chain^38^.

By incorporating LPO with the GO-SA, the FTIR results recorded several variations between the spectrum of GO-SA and GO-SA-LPO composites. Where after coating LPO on the surface of GO-SA nanomaterial, a wide, intense band at 3207 cm^−1^ (revealed to physically adsorbed water molecules) was appeared, which might due to the H-bond between the water molecules and different function groups in the LPO molecules. Moreover, band 2915 cm^−1^ due to C-H stretching related to the carbohydrate groups of the LPO molecules. The disappearance of the peak at 1726 cm^−1^ in the GO-SA-LPO composite may be due to the consumption of carboxylate groups in the interaction with LPO. The observation of new peaks at 1541 and 1458 cm^−1^ referred to N–H and s C-N stretching, respectively^39^.

Based on the data obtained by the FTIR, the GO-SA composite does not possess an adsorbed water peak; thus, only 3% of its weight was lost at T = 176 ^o^C. On the other hand, the GO-SA-LPO composite presents weight loss (15%, 95 ^o^C) due to the high amount of physically absorbed water molecules due to the coating of LPO. The GO-SA nanocomposite will show a higher pyrolysis rate in the next stages than the modified GO-SA-LPO nanomaterials. This can be explained by the loading of LPO on the surface of GO-SA composite, which will generate more stable bonds such as amide and C-N bonds, requiring more pyrolysis temperature to break down. Hydrodynamic size and zeta potential modifications of the prepared GO-SA composite and the modified GO-SA-LPO composite were employed to affirm the enzyme assembly on the surface of the prepared GO-SA composite. The hydrodynamic size and zeta potential of the prepared GO-SA and the modified GO-SA-LPO composites were assessed and compared. The obtained results showed that there was a significant difference in the hydrodynamic size of GO-SA before (4797 nm) and after the immobilization process (746 nm). The zeta potentials of the prepared GO-SA and the modified GO-SA-LPO composites were − 13 and − 12 mV, respectively. The negative charge of the GO-SA surface (-13 mV) could be mainly due to the carboxylate groups of alginates. Following the incorporation of LPO with composite, the zeta potential of the modified GO-SA-LPO composite was decreased to -12 mV since LPO is a cationic enzyme (isoelectric pH is 9.6) and displays the positive electric charge at pH 6.8^39^. This result confirms a successful coating of the enzyme on the surface of the prepared GO-SA composite and forms the modified GO-SA-LPO composite.

Colon tumor is one of the most frequent tumors that affect both females and males, and it is the second most common tumor after lung tumors in Western and developing countries^40^. Global statistics demonstrate that although the prevalence and the mortality rate of colon cancer have been decreased successfully among people aged over 50 owing to effective cancer screening tools, an increase of about 1.8% has been diagnosed in younger patients. It is expected that the average prevalence of colon cancer may increase by about 107.1% in young patients aged 20–34 years by 2030^41,42^. Although many advances in systematic therapy have progressed, such as targeted therapy, immunotherapy, and chemotherapy, most colorectal metastatic patients die of their disease, and about 33% of patients have distant metastasis^37^. These gloomy statistics represent a challenging goal for scientists and clinicians to explore new anti-colorectal cancer therapies to overcome the limitations of the currently existing drugs. Recent data show that colorectal cancer is an immune-responsive disease, and many groups of patients with progressive colorectal disease achieved long-term recovery with an immunotherapy approach^43^. Because LPO did not show any cytotoxicity to the surrounding normal cells and tissues of colorectal organs, this important enzyme should be considered in further in vitro and in vivo trials as the possibility of using it as a new systemic immunotherapy for metastatic colorectal cancer treatment.

Moreover, our previous data showed that the nanoformulation of LPO with LF was superior in their selective apoptosis-mediating antitumor activity against several types of cancer cells, including colon cancer than the free forms of these proteins^27^. Recent in vitro investigation demonstrated that nanocombinations of the purified bovine LPO and lactoferrin (LF)-coated Cu and Fe nanometals provoked their apoptotic effect *via* cell cycle arrest mechanism with up-regulating proapoptotic genes (p53) and downregulating oncogenes (Bcl-2) in the treated MCF-7 breast cancer cells^26^. Moreover, using a bone resorption model, LPO enhanced the suppression of NF-κB expression in an in vitro study^44^. Another study indicated that after treatment with the nanoformulated LPO and LF on chitosan NPs, there was a considerable suppressed various pro-inflammatory markers, including TNF-α, IL-6, VEGF, and NF-κB in the treated breast MCF-7 and MDA cancer cells. TNF-α is a key cytokine in inflammatory responses, besides its function as an endogenous tumor promoter^45^. A recent study has reported that the anticancer activity of immobilized LF on graphene-based composites contributed to arresting the G1/S cell cycle, decreasing the expression levels of pro-Caspase-3 and phospho-AKT levels and up-regulating the level of cleaved caspase 3 in the treated cells^46^. The authors demonstrated that more anticancer effect of the LF immobilized graphene-based composite compared to free LF was associated with its long circulation time owing to stimulation of protein stability, more cellular internalization, and higher cytotoxic effect, which in agreement with our findings in the present work.

Furthermore, the anticancer activity of graphene-based composites seems to be dependent on the type of treated cancer cells, functional groups, and size of the prepared NPs may be effective on its effectiveness. Furthermore, another previous study demonstrated that the purified bovine LPO showed a potent anticancer activity through its contribution to the apoptosis-dependent antineoplastic effect of iodine or iodide in a DAMB-treated mammary gland tumor in rat model^47^. The authors found that the treated DMBA-induced breast cancer in a rat model with 0.1% molecular iodine/potassium iodide induced the expression of LPO, which contributes to the antineoplastic effect, inducing apoptosis through PPARγ/caspases and preventing estrogen-induced DNA adducts in pre-cancer and cancerous cells. Our previous findings also proved that the synergistic effect of LF and LPO nanocombination was exerted via p53-mediated apoptotic mechanism in treated cancer cells^27^. The synergistic index of LF and LPO (CI < 1) was calculated to be less than 1 for its antitumor effect toward all treated cancer cell lines, which refers to their potent synergistic effect. This interesting previous study also revealed that LF and LPO nanocombination induced cell cycle arrest in all treated tumor cells^27^.

## Conclusion

The results presented here proved the successful nanoformulation of the purified LPO with alginate-modified graphene oxide, forming the modified GO-SA-LPO composite with potent anticancer activity against colon cancer cell lines and high safety on normal cells. The potent anti-colon cancer effect of the modified GO-SA-LPO composite could be attributed to its ability to provoke apoptosis pathway with up-regulating of proapoptotic genes and downregulating of oncogenes in the treated colon cancer cells compared to free LPO or the prepared GO-SA composite. We strongly believe that the capability of the modified GO-SA-LPO composite to target colon tumors with a high degree of selectivity may offer an increase in the lifetime survival of LPO with dose reduction of treatment and low toxicity. These findings greatly support future uses of the modified nanocombination as a promising therapeutic candidate for fighting different types of cancer with high safety on normal cells.

## Supplementary Information

Supplementary Material 1.

## Data Availability

All data generated or analysed during this study are included in this published article as Supplementary Material and supplementary information files.
