# Supplementary material for: Alginate-modified graphene oxide anchored with lactoperoxidase as a novel bioactive nanocombination for colorectal cancer therapy
Source: Sci Rep. 2024 Oct 22;14:24804. doi: 10.1038/s41598-024-74604-0 (PMC11496692; doi:10.1038/s41598-024-74604-0)
Supplement: Supplementary file 1 — Supplementary Material 1. [file 41598_2024_74604_MOESM1_ESM.docx]

**Supplementary Material**

**Alginate-modified graphene oxide anchored with lactoperoxidase as a novel bioactive nanocombination for colorectal cancer therapy**

**AbdElAziz A.Nayl^1,*^, Esmail M. El-Fakharany^2^, Ahmed I. Abd-Elhamid^3^ , Wael A. A. Arafa^1^, Ahmed H. Alanazi^1^, Ismail M.Ahmed^1^, Mohamed A. Abdelgawad ^4^, Ashraf A. Aly** ^5^**, Stefan Bräse**^6^**^,^**^7^**^,*^**

^1^ Department of Chemistry, College of Science, Jouf University, Sakaka, Aljouf 72341, Saudi Arabia.

^2^ Protein Research Department, Genetic Engineering and Biotechnology Research Institute GEBRI, City of Scientific Research and Technological Applications (SRTA city), New Borg El-Arab, Alexandria 21934, Egypt. [esmailelfakharany@yahoo.co.uk](mailto:esmailelfakharany@yahoo.co.uk)

^3^ Composites and Nanostructured Materials Research Department, Advanced Technology and New Materials Research Institute, City of Scientific Research and Technological Applications (SRTA-City), New Borg Al-Arab, Alexandria 21934, Egypt; [ahm_ch_ibr@yahoo.com](mailto:ahm_ch_ibr@yahoo.com).

^4^Department of Pharmaceutical Chemistry, College of Pharmacy, Jouf University, Sakaka, Al Jouf 72341, Saudi Arabia; [mhmdgwd@ju.edu.sa](mailto:mhmdgwd@ju.edu.sa).

^5^Chemistry Department, Faculty of Science, Organic Division, Minia University, El-Minia 61519, Egypt; [ashrafaly63@yahoo.com](mailto:ashrafaly63@yahoo.com)

^6^ Institute of Organic Chemistry (IOC), Karlsruhe Institute of Technology (KIT), Kaiserstrasse 12, 76131 Karlsruhe, Germany

^7^Institute of Biological and Chemical Systems – Functional Molecular Systems (IBCS-FMS), Kaiserstrasse 12, 76131 Karlsruhe, Germany

* Correspondence: [aanayel@ju.edu.sa](mailto:aanayel@ju.edu.sa) or [aanayl@yahoo.com](mailto:aanayl@yahoo.com) (A.A.N.); [stefan.braese@kit.edu](mailto:stefan.braese@kit.edu) (S.B.)

**2. Materials and Methods**

**2.1. Chemicals, materials, and instruments**

All the chemicals and materials used were analytical grade and used without further treatment. TEOS (99%, Across), ethanol absolute (Sigma-Aldrich), KMnO_4_ (99%, Long live), H_2_SO_4_ (95–97%, Riedel deHaen), sodium alginate (Alpha Chemika, analytical reagent), H_2_O_2_ (36%, Pharaohs Trading and Import), HCl (30%, El Salam for Chemical Industries), and graphite (200mesh, 99.99%, Alpha Aesar), Analytical balance (CP 2245, Sartorius, USA.), Hot plate stirrer (IKA, C-MAG HS7, IKA®-Werke GmbH & Co. KG, Germany), Centrifuge, (Mikro 220R, Hettich, UK.).

**2.2. Purification of bovine milk lactoperoxidase (LPO)**

Bovine LPO was extracted from bovine skimmed milk. first the milk fat was removed by centrifugation at high speed for 1/2hr. Thereafter, decatenated by lowering the pH to 4.2 with 1 M HCl [19]. After dialysis, the obtained skimmed milk against 50 mM phosphate buffer, pH 7.2, and about 100 mg protein was applied into Mono S 5/50 GL column pre-equilibrated with 50 mM phosphate buffer, pH 7.2. The bound proteins were eluted with a stepwise gradient of 0.0 to 1.0 M NaCl prepared in 50 mM phosphate buffer, pH 7 at a 1 mL/min flow rate and 4 mL fractions. All fractions of LPO were concentrated and desalted separately by a centricon ultrafiltration cell (30 kDa MWCO). About 30 mg protein was applied into a Sephacryl S100 column (5×150 mm, GE Health care, Sweden) pre-equilibrated with 50 mM phosphate buffer, pH 7.2, containing 150 mM NaCl. The purified LPO was eluted with the same buffer at a 1 mL/min flow rate and 4 mL fractions. The homogeneity and purity of LPO were evaluated by 12% SDS-PAGE, and all fractions containing the purified LPO were pooled, dialyzed, lyophilized, and kept at −20 °C until further uses.

**2.7.2. Cytotoxicity of the modified NPs**

The antitumor properties of the functionalized GO-SA composite and the modified GO-SA-LPO composite against both normal and tumor cell lines were investigated using MTT (3-[4, 5-Dimethylthiazol]-2, 5-Diphenyltetrazolium bromide) assay as described in our lab by [22]. In Brief, all cell lines (1.0×10^4^/well) were seeded in three sterile 96-well microplates overnight at 37°C in supplemented media with 10% FBS. Both normal and tumor cell lines were exposed to the GO-SA composite and the modified GO-SA-LPO composite at different concentrations of 12.5, 25, 50, 100, 200, and 400 μg/ml in triplicates. After incubation for 48 h, the treated cells were washed three times with fresh media, and 200 μl from 0.5 mg/ml MTT solution was added to each well, and the cells were incubated for 2-3 h to allow MTT metabolization by viable cells. 200 μl of dimethylsulfoxide (DMSO) was added to each well for dissolving formazan crystals, and the absorbance was measured at 570 nm using a microplate reader. Untreated healthy cells were included as a positive control. The IC_50_ value (half maximal inhibitory concentration of each of the prepared NPs that kill 50% of the treated cells) was determined by the GraphPad Instate software 6.0 and values of selectivity index (SI), which defined as the ratio of IC_50_ of normal cell line versus each tumor cell line was calculated **[21]**. Furthermore, the morphology of the treated Caco-2 cells was visualized under a phase contrast microscope (Olympus, Japan) at concentrations of 50, 100, and 200 μg/ml compared to untreated cells.

**2.8. Effect of the modified GO-SA-LPO composite** **on gene expression**

The effect of the prepared GO-SA composite and the modified GO-SA-LPO composite on the expression level of tumor suppressor gene (p53) and oncogene (Bcl-2) was determined in human Caco-2 and HCT-166 cells using the qPCR method. Total RNAs were extracted from untreated and treated Caco-2 and HCT-166 cells with IC_50_ doses for 48 h. Total RNAs were extracted using the Gene JET RNA Purification Kit protocol (Thermo Scientific, USA). Real-time PCR (qPCR) was performed using a master mix of SYBR green kit using specific primers (Forward/Reverse) as follows: 5′-TAACAGTTCCTGCATGGGCGGC-3′/5′-AGGACAGGCACAAACACGCACC-3′ for p53 gene and 5′-TCCGATCAGGAAGGCTAGAGTT-3′/5′-TCGGTCTCCTAAAAGCAGGC-3′ for Bcl-2 gene. The change in the expression level for each gene before and after treatment was determined using the equation of 2^-ΔΔCT^.
